# Influence of serum uric acid on bone mineral density across body mass index categories in men with type 2 diabetes: a cross-sectional study

**DOI:** 10.1186/s12891-025-09169-8

**Published:** 2025-11-13

**Authors:** Yujun Qin, Chunmiao Huang, Wenhe Wei, Yi Zhang

**Affiliations:** 1 Department of General Practice, The First Affiliated Hospital of Guangxi Medical University, Guangxi Zhuang Autonomous Region, Nanning, P.R. China; 2 Department of Endocrinology, The People’s Hospital of Hechi, Guangxi Zhuang Autonomous Region, Hechi, P.R. China

**Keywords:** Serum uric acid, Bone mineral density, Diabetes, Body composition, Body mass index

## Abstract

**Background:**

The impact of different serum uric acid(SUA) concentrations on bone mineral density(BMD) varied across different BMI categories, which might provide useful insights for clinical uric acid-lowering treatments.

**Purpose:**

To explore whether SUA was associated BMD across BMI categories in men aged ≥ 50 years with type 2 diabetes (T2D).

**Methods:**

The participants were divided into six groups according to SUA levels. Analyses were adjusted for the covariates including weight(Wt), total lean mass(TLM), total fat mass(TFM), total fat ratio(TFr), A/G and a range of other baseline laboratory findings.

**Results:**

SUA was associated with total lumbar BMD(TLBMD) (*p* = 0.013). In the BMI(24–27.999 kg/m^2^) group, SUA was significantly associated with TLBMD (*p* = 0.02). There was no correlation between SUA and whole-body BMD, as well as femoral neck BMD and total hip BMD. In the BMI < 24 kg/m^2^ group, the highest TLBMD value was observed when SUA levels ranged from 420 µmol/L to 479 µmol/L. While BMI:24–27.999 kg/m^2^, the highest TLBMD value was showed in the SUA(360-419 μmol/L) group based on adjusted models. The SUA(300-360 μmol/L) group exhibited the highest TLBMD value among the patients with BMI ≥ 28 kg/m^2^.

**Conclusion:**

SUA was correlated with TLBMD in T2D men aged ≥ 50 years. Among patients with BMI 24–27.999 kg/m^2^, the SUA concentrations between 360–419 μmol/L might have a protective effect on TLBMD.

## Background

Type 2 diabetes (T2D) and osteoporosis are common chronic diseases that have a high prevalence in the aging population [1]. Both conditions serve as independent predictors of overall mortality[2]. The risk of fractures increases in individuals with diabetes due to secondary osteoporosis [3]. It is crucial to actively screen and manage osteoporosis in patients with diabetes [4]. Osteoporosis in men is an increasingly serious yet poorly recognized public health issue [5]. Out of the 8.9 million fractures caused by osteoporosis worldwide each year, 20%−25% occured in men [6]. It was concerning that only 2.1% individuals were diagnosed with and receive appropriate treatment for osteoporosis [7]. Approximately 25% of men aged 50 and above experienced bone fractures due to osteoporosis, with poorer prognosis observed in men [8]. The factors contributing to worse outcomes in men remained elusive; However, advanced age and comorbidities seemed to be associated with increased mortality risk within the first year following the fracture [9]. 50% to 80% of men had experienced secondary osteoporosis [10]. It was crucial to exclude secondary causes as treatment for these patients typically began with addressing the underlying condition [11]. Elevated serum uric acid (SUA) levels had been shown to significantly increase in conditions such as obesity, gout, T2D, and metabolic syndrome [12]. This elevation appeared to contribute to the development of comorbidities associated with these diseases [13]. According to a retrospective study [14], the results revealed a positive correlation between body mass index (BMI), SUA, high-density lipoprotein cholesterol(HDL-C), and estimated glomerular filtration rate (eGFR) with BMD (*p* < 0.05). However, a cross-sectional analysis of 250 T2D patients without comorbidities revealed that triglycerides(TG), total cholesterol(TC), HDL-C, HbA1c and SUA had a significant association with the lowest T-score of BMD or the risk of osteoporosis [15]. The correlation between SUA and osteoporosis had been explored in recent research. According to a retrospective cross-sectional study, a positive correlation was observed between SUA levels and BMD of the lumbar, femoral neck, and total femur [16]. According to research, there was a significant correlation between higher levels of SUA and higher BMD [17]. SUA may have a protective effect on bone metabolism, possibly due to its antioxidant properties [18]. It had been discovered that under SUA normal levels, there was a correlation between SUA and increased BMD, which can help prevent fractures [19]. However, in cases of hyperuricemia or gouty arthritis, elevated SUA levels can increase the risk of fractures, as oxidative stress and inflammatory cytokines can enhance bone resorption and decrease bone formation [20]. According to an analysis conducted on patients with osteoporosis who underwent surgery, SUA levels were positively correlated with BMD independently; in normal and low weight patients, SUA levels < 296 μmol/L may have a protective effect on BMD, while SUA levels > 296 μmol/L had no effect on BMD [21]. Recent research had also focused on the impact of hypouricemia, and multiple studies had indicated that abnormally low SUA levels may lead to potential destructive effects [22, 23]. According to some research, a significant positive correlation had been observed between SUA, BMI, and BMD in both the lumbar and hip among men with diabetes [24]. However, according to another cross-sectional study, the bone-protective effect of SUA was mediated by age and gender; SUA was only positively correlated with BMD in young men and elderly women with T2D [25]. The association between SUA and BMD remained controversial in patients with T2D.

Currently, there was still no definite conclusion on the relationship between SUA and BMD based on numerous epidemiological and experimental studies. Our purpose was to investigate the correlation between SUA and BMD in T2D men aged ≥ 50 years. BMI and body composition may mediate the relationship between SUA and BMD. Thus, we analyzed body composition using Whole-body dual-energy X-ray absorptiometry and examined indicators including whole body lean mass, fat mass, fat ratio, and android/gynoid ratio(A/G) to comprehensively analyze the relationship. By exploring the optimal SUA levels that were beneficial for BMD across different BMI groups, we aimed to provide a basis for clinical treatment and control of SUA within the appropriate range. This study also laid the foundation for further expanding the sample size and conducting more extensive research.

## Materials and methods

### Study design and subjects

This was a prospective cross-sectional study. The study was approved by the Ethics Committee of The People's Hospital of Hechi (approval No. Z-M20221849), and written consent was obtained from all participants in accordance with the ethical principles of the Declaration of Helsinki.

Inclusion criteria: 1. A confirmed diagnosis of Type 2 Diabetes (T2D) based on the "Standards of Medical Care for Type 2 Diabetes in China" [26], or patients receiving treatment for diabetes (such as medications or insulin injections) following a medical diagnosis. 2. The ability and willingness to provide informed consent. 3. Males aged 50 years or older. Exclusion criteria: 1. Intellectual disabilities. 2. Malnutrition resulting from severe organic disease. 3. Patients currently undergoing urate-lowering therapy (ULT) or treatment with anti-osteoporosis medications. 4. Use of calcium or vitamin D supplements. 5. Presence of metallic objects within the body. 6. Severe acute inflammatory diseases or chronic medical/neurological conditions that could significantly influence biochemical Markers. 7. Coexisting conditions such as fractures, bone tumors, bone tuberculosis, or other bone-related pain disorders. 8. Long-term use of hormone-based medications or other substances that can alter bone metabolism.

**Fig. 1:**
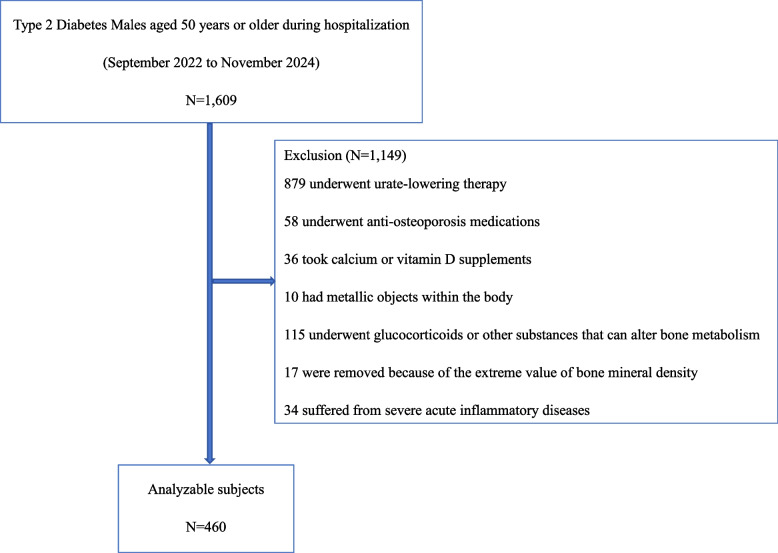
Study flow chart

The present study enrolled consecutive hospitalized primary T2D Males with definitive diagnoses. 1,609 hospitalized primary T2D Males aged ≥ 50 years were evaluated for study inclusion (Fig. 1). Patients were excluded according to exclusion criteria. Finally, a total of 460 subjects were recruited over a 26-month period spanning from September 2022 to January 2025 (originally anticipated to conclude by November 2024). Data collection has been completed as of January 2025.

### Assessment

All participants underwent an assessment by a senior expert in endocrinology.

Patients were stratified into six groups based on SUA clinical cutoffs [27] (G1 < 240 μmol/L, G2: 240-299 μmol/L, G3: 300-359 μmol/L, G4: 360-419 μmol/L, G5: 420-479 μmol/L, and G6 ≥ 480 μmol/L).

During the initial evaluation, measurements of weight, height, waist circumference, and hip circumference were taken. Body Mass Index (BMI) was calculated using the formula: BMI = weight (kg)/height (m^2^). Patients were categorized into three groups based on BMI [28] as follows: BMI < 24 kg/m^2^, BMI 24–27.999 kg/m^2^, and BMI ≥ 28 kg/m^2^.

Additionally, socio-demographic and clinical data were recorded, including age, gender, marital status, occupation, age at onset, and disease duration.

Whole-body composition was assessed using dual-energy X-ray absorptiometry (DXA) [29, 30] with a fan-beam densitometer (Discovery-W, Hologic Inc., Bedford, MA, USA) to determine total fat mass (TFM), total lean mass (TLM), total fat ratio (TFr), trunk-to-limb fat ratio (TLfr), A/G ratio, and BMD. All measurements were performed by certified radiological technicians.

### Covariate analyses

Blood samples were collected between 6:00 a.m. and 8:00 a.m. after an overnight fast, at the time of hospital admission. The following parameters were measured using an automated analyzer (Hitachi Automatic Analyzer 7600, Tokyo, Japan): total cholesterol (TC), triglycerides (TG), low-density lipoprotein cholesterol (LDL-C), fasting blood glucose (FBG), calcium levels, serum creatinine (SCr), and alkaline phosphatase (ALP) levels. Serum uric acid (SUA) was quantified using the Beckman Synchron LX20 system. Glycated hemoglobin (HbA1c) levels were determined via high-performance liquid chromatography with an automated glycohemoglobin analyzer (Tosoh HLC-723G7, Tokyo, Japan). Fasting insulin (FINS) and C-peptide levels were assessed using immunoradiometric assays. Additionally, serum 25-hydroxyvitamin D (25(OH)D) levels were measured using radioimmunoassay with a gamma counter (1470 WIZARD, PerkinElmer, Turku, Finland).

### Statistical analyses

Descriptive statistics were conducted for the entire sample. Continuous demographic, laboratory, and clinical variables were presented as means with standard deviations (SD). BMD across different groups, categorized by qualitative variables, was compared using one-way analysis of variance (ANOVA). For data that did not follow a normal distribution or had unequal variances, non-parametric methods were employed. Continuous variables were compared using independent t-tests for normally distributed data, and Mann–Whitney U tests for non-normally distributed data. Univariate analyses were performed to examine the associations between patient characteristics and BMD in individuals with T2D. To investigate the independent relationship between SUA levels and BMD, generalized estimating equations (GEE) were utilized, adjusting for relevant covariates [31]. Weighted multivariate linear regression models were applied to assess the association between SUA and BMD across three distinct models: Model 1 (unadjusted), Model 2 (adjusted for BMI), and Model 3 (adjusted for all covariates). To evaluate the robustness of the findings and potential variations across patient subgroups, subgroup analyses were conducted by stratifying patients based on specific covariates, with likelihood ratio tests (LRT) used to assess interactions and effect modifications. SUA was categorized based on clinically relevant cutoff points. Subgroup analyses and interaction tests were performed based on BMI and SUA levels. All statistical analyses were carried out using the Statistical Package for the Social Sciences (SPSS) software (version 26.0). A significance level of *p* < 0.05 was considered statistically significant.

## Results

### Patients’ characteristics

Baseline characteristics for patients (men aged ≥ 50 years) with T2D hospitalized (*n* = 460) in the SUA groups were summarized in Table 1.

**Table 1:** Patient characteristics based on SUA groups

Characteristics	Total	Mean ± SD						*P*
		**G1(< 240 μmol/L)**	**G2(240-299 μmol/L)**	**G3(300-359 μmol/L)**	**G4(360-419 μmol/L)**	**G5(420-479 μmol/L)**	**G6(≥ 480 μmol/L)**	
N	460	41	73	97	95	76	78	
Age, years	61.283 ± 7.777	61.268 ± 7.301	60.247 ± 7.27	61.474 ± 7.178	62.253 ± 8.713	60.013 ± 7.267	62.077 ± 8.402	0.34
TLBMD, T-value	−1.08 ± 1.19	−1.402 ± 0.871	−1.414 ± 1.135	−0.997 ± 1.255	−0.971 ± 1.214	−0.822 ± 1.231	−1.087 ± 1.167	0.013
FNBMD, T-value ^m^	−1.2	−1.3	−1.4	−1.1	−1.1	−1.1	−1.3	0.249
THBMD, T-value ^m^	−0.8	−1	−1.1	−0.8	−0.7	−0.8	−0.8	0.354
TBMD, g/cm^2^	1.11 ± 0.096	1.094 ± 0.08	1.098 ± 0.092	1.106 ± 0.091	1.131 ± 0.111	1.124 ± 0.096	1.098 ± 0.089	0.074
TLM, g	42,625.991 ± 6050.021	39,982.054 ± 7549.09	42,099.574 ± 6312.69	42,190.333 ± 5105.723	42,726.101 ± 5241.953	43,765.838 ± 5942.992	43,817.657 ± 6627.141	0.011
TFM, g	20,588.745 ± 6064.288	18,201.078 ± 5207.567	19,335.558 ± 6267.696	19,692.629 ± 5415.428	20,218.264 ± 5825.228	22,440.075 ± 6093.185	22,778.422 ± 6348.52	< 0.001
TFr, %	30.905 ± 4.998	29.04 ± 5.369	29.803 ± 5.099	30.286 ± 4.823	30.572 ± 4.971	32.374 ± 4.886	32.662 ± 4.278	< 0.001
Trunk/LegsFr, %	1.194 ± 0.136	1.14 ± 0.117	1.163 ± 0.135	1.205 ± 0.129	1.202 ± 0.119	1.197 ± 0.139	1.227 ± 0.16	0.007
Trunk/LimbFr, %	1.445 ± 0.216	1.387 ± 0.206	1.391 ± 0.235	1.456 ± 0.204	1.427 ± 0.179	1.471 ± 0.227	1.509 ± 0.23	0.006
A/G, %	1.225 ± 0.177	1.18 ± 0.129	1.192 ± 0.177	1.217 ± 0.188	1.242 ± 0.176	1.245 ± 0.211	1.252 ± 0.142	0.115
H(cm)	165.336 ± 5.568	164.439 ± 5.32	165.973 ± 5.538	165.119 ± 5.494	165.211 ± 5.023	165.382 ± 5.697	165.59 ± 6.358	0.796
Wt(kg)	67.572 ± 11.506	63.373 ± 9.679	65.918 ± 12.542	66.247 ± 10.639	67.303 ± 10.346	70.48 ± 11.422	70.468 ± 12.783	0.003
BMI, kg/m^2^	24.685 ± 3.82	23.456 ± 3.625	23.812 ± 3.552	24.252 ± 3.392	24.689 ± 3.938	25.775 ± 4.043	25.618 ± 3.918	0.001
Waistline, cm	90.128 ± 10.414	87.037 ± 9.244	88.897 ± 12.835	89.216 ± 9.598	90.153 ± 9.858	91.842 ± 10.04	92.341 ± 10.045	0.051
Hipline, cm	95.067 ± 7.806	93.744 ± 6.862	95.151 ± 10.355	93.753 ± 6.686	94.755 ± 7.038	96.289 ± 8.02	96.505 ± 7.271	0.125
SUA, μmol/L	378.389 ± 115.659	192.512 ± 40.237	270.452 ± 16.276	329.371 ± 19.585	388.126 ± 17.417	448.487 ± 17.904	557.91 ± 86.077	< 0.001
TC, mmol/L	5.055 ± 1.355	4.853 ± 1.148	5.028 ± 1.442	4.936 ± 1.386	5.208 ± 1.408	4.959 ± 1.242	5.236 ± 1.372	0.462
TG, mmol/L ^m^	1.66	1.22	1.35	1.53	1.715	1.975	2.3	< 0.001
LDLc, mmol/L	2.708 ± 0.96	2.649 ± 0.913	2.755 ± 1.09	2.571 ± 0.948	2.818 ± 0.928	2.664 ± 0.846	2.774 ± 1.011	0.543
ALP, U/L	84.223 ± 36.717	82.415 ± 28.215	88.863 ± 45.297	88.34 ± 43.734	81.745 ± 34.031	78.392 ± 27.568	84.231 ± 32.62	0.445
SCr, μmol/L ^m^	81	71	68	74	82	94	97.5	< 0.001
FBG, mmol/L ^m^	8.59	9.33	9.56	9.38	8.015	7.865	7.635	< 0.001
2hPG, mmol/L	17.592 ± 5.594	19.183 ± 5.677	19.327 ± 5.628	18.001 ± 5.264	17.046 ± 5.582	16.322 ± 5.131	16.486 ± 5.875	0.002
HbA1c, % ^m^	9.5	10.25	11.1	10.7	8.95	8.5	8.4	< 0.001
FINS, umol/l ^m^	8.08	10.545	7.77	6.85	7.545	9.48	8.915	0.179
2hINS, umol/l ^m^	24.355	20.885	14.63	19.58	25.38	31.77	29.43	< 0.001
FC-peptide, nmol/L ^m^	1.965	1.47	1.35	1.59	2.08	2.05	2.645	< 0.001
2hC-peptide, nmol/L ^m^	4.6	3.735	3.61	3.72	4.95	5.785	5.91	< 0.001
Calcium levels, mmol/L	2.262 ± 0.154	2.229 ± 0.151	2.244 ± 0.181	2.252 ± 0.121	2.288 ± 0.156	2.27 ± 0.164	2.268 ± 0.151	0.274
25(OH)D levels, ng/mL	30.118 ± 11.196	31.241 ± 10.584	31.346 ± 11.179	29.63 ± 11.01	29.49 ± 11.955	31.096 ± 11.178	28.84 ± 10.927	0.651
Duration of T2D, months ^m^	60	36	36	48	72	36	84	0.079

*Abbreviations*: *SUA* serum uric acid, *SD* standard deviation, *G group* N number, *TLBMD* total lumbar bone mineral density, *FNBMD* femoral neck bone mineral density, *THBMD* total hip bone mineral density, *TBMD* whole body bone mineral density, *TLM* total lean mass, *TFM* total fat mass, TFr total fat ratio, *Fr* fat ratio, *A/G* android/gynoid ratio, *H* height, *Wt* weight, *BMI* body mass index, *TC* total cholesterol, *TG* triglyceride, *LDL-c* low-density lipoprotein cholesterol, *ALP* alkaline phosphatase, *SCr* serum creatinine, *FBG* fasting blood glucose, *HbA1c* glycosylated hemoglobin type A1C, *FINS* fasting serum insulin, *25(OH)D* 25-hydroxy vitamin D, *T2D* type 2 diabetes

^m^ median, *P*-value < 0.05 was considered statistically significant

The average age was 61.75 ± 7.637 years. The mean TBMD for these patients was 1.11 ± 0.096 g/cm^2^, while the mean SUA was 378.389 ± 115.659 μmol/L in the overall patients. Patients were stratified into six groups based on SUA clinical cutoffs (G1 < 240 μmol/L, G2: 240-299 μmol/L, G3: 300-359 μmol/L, G4: 360-419 μmol/L, G5: 420-479 μmol/L, and G6 ≥ 480 μmol/L), and differences in TLBMD, TLM, TFM, TFr, Trunk/LegsFr, Trunk/LimbFr, Wt, BMI, TG, SCr, FBG, 2hPG, HbA1c, 2hINS, FC-peptide and 2hC-peptide were evident among these groups. The correlation between SUA and TLBMD was observed (*P* = 0.013), whereas no correlation was found between SUA and TBMD (*P* = 0.074), FNBMD (*P* = 0.249), or THBMD(*P* = 0.354).

### Exploration of the association between SUA and TLBMD

Three models were used to examine the relationship between SUA and TLBMD in T2D patients (Table 2). A relationship between SUA and TLBMD was evident in the unadjusted Model 1 (*β* = 0.001, *P* = 0.013, R^2^ = 0.014). Model 2, which was adjusted for BMI, exhibited no association (*β* = 0.001, *P* = 0.106, R^2^ = 0.068). There was no evident for Model 3 (*β* = 0.001, *P* = 0.208, R^2^ = 0.148) following adjustment for BMI, LDL, 25(OH)D, H, ALP, age, FINS, FPG, Ca, duration of T2D, TG, Trunk/LimbFr, 2hCpeptide, 2hPG, SCr, AG, FCpeptide, 2hINS, HbA1c, TFr, Hipline, TC, TLM, Trunk/LegsFr, Waistline, TFM, and Wt.

**Table 2:** Association between SUA levels and TLBMD in different models

		**Total**	**BMI, kg/m**^**2**^		
			** < 24**	**24–27.999**	** ≥ 28**
Model 1	β	0.001	0.000	0.003	−0.001
	*P*	0.013	0.724	0.002	0.646
	R^2^	0.014	0.001	0.070	0.002
	adj R^2^	0.012	−0.004	0.063	−0.009
Model 2	β	0.001	0.000	0.003	−0.001
	*P*	0.106	0.997	0.002	0.656
	R^2^	0.068	0.060	0.074	0.003
	adj R^2^	0.063	0.050	0.061	−0.021
Model 3	β	0.001	0.000	0.003	−0.001
	*P*	0.208	0.586	0.001	0.347
	R^2^	0.148	0.194	0.278	0.508
	adj R^2^	0.088	0.063	0.095	0.270

Model 1 No adjustment

Model 2 Adjusted for BMI

Model 3 Adjusted for BMI, LDLc, 25(OH)D, H, ALP, age, FINS, FPG, Ca, duration of T2D, TG, Trunk/LimbFr, 2hCpeptide, 2hPG, SCr, AG, FCpeptide, 2hINS, HbA1c, TFr, Hipline, TC, TLeanMass, Trunk/LegsFr, Waistline, TFM, and Wt

*P* < 0.05 was considered statistically significant

The participants were divided into three groups according to the obesity diagnostic criteria based on BMI: BMI < 24 kg/m^2^, BMI 24–27.999 kg/m^2^, and BMI ≥ 28 kg/m^2^. In the BMI 24–27.999 kg/m^2^ group, SUA was significantly associated with TLBMD (*P* = 0.02). After adjusting for BMI (Model 2, *P* = 0.02) and other confounding factors (Model 3, *P* = 0.01), this association remained independent. However, no such association between SUA and TLBMD was observed in the BMI < 24 kg/m^2^ and BMI ≥ 28 kg/m^2^ groups (Table 2).

### The relationship between SUA and TLBMD under BMI groups

**Fig. 2:**
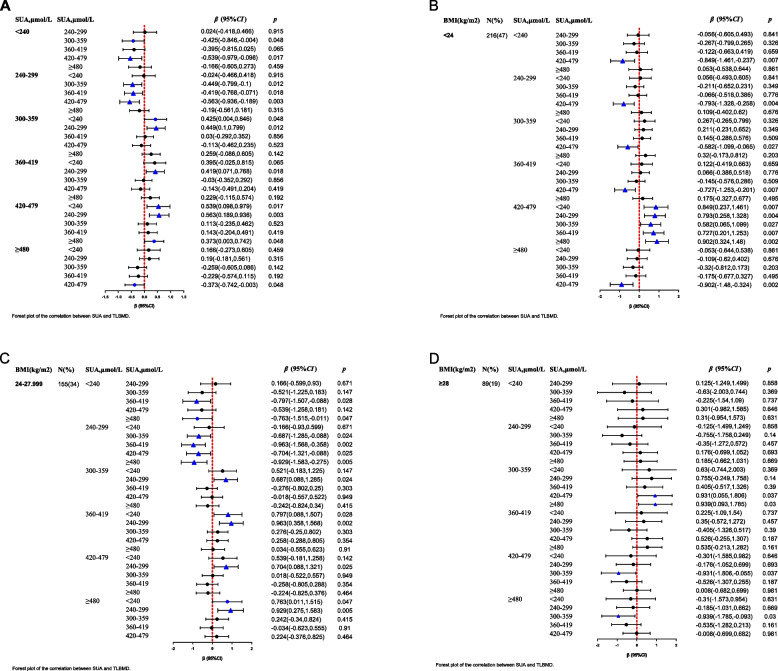
Forest plot of the correlation between SUA and TLBMD

In the SUA grouping, the TLBMD value in G5 was the highest, significantly increased compared to the TLBMD values in the G1 (*β* = 0.539, 95% *CI*: 0.098 to 0.979, *P* = 0.017), G2 (*β* = 0.563, 95% *CI*: 0.189 to 0.936, *P* = 0.003), and G6 (*β* = 0.373, 95% *CI*: 0.003 to 0.742, *P* = 0.048) groups (Fig. 2A). Further comparison by BMI groups revealed that in the BMI < 24 kg/m^2^ group, the TLBMD values significantly decreased in the groups with SUA levels below 420 µmol/l or above 479 µmol/l (Fig. 2B). In the BMI 24–27.999 kg/m^2^ group, the TLBMD value was highest when SUA levels ranged from 360 µmol/l to 419 µmol/l, which was significantly higher than the TLBMD values in the G1 (*β* = 0.797, 95% *CI*: 0.088 to 1.507, *P* = 0.028) and G2 (*β* = 0.963, 95% *CI*: 0.358 to 1.568, *P* = 0.002) groups(Fig. 2C). In the BMI ≥ 28 kg/m^2^ group, the TLBMD values decreased as SUA levels increased, with the TLBMD value in the group with SUA levels above 420 µmol/l(G5、G6) being significantly lower than that in the G3 group (Fig. 2D).

## Discussion

Our study demonstrated a significant positive correlation between serum uric acid (SUA) and total lumbar bone mineral density (TLBMD) specifically in men (BMI 24–27.999 kg/m^2^) with type 2 diabetes (T2D), independent of multiple confounders including LDL, 25(OH)D, age, HbA1c, and body composition parameters. This association was absent in both BMI < 24 kg/m^2^ and BMI ≥ 28 kg/m^2^ individuals. Crucially, optimal SUA levels for TLBMD differed across BMI categories: 420–479 μmol/L in BMI < 24 kg/m^2^ group, 360–419 μmol/L in BMI 24–27.999 kg/m^2^ group, and 300–360 μmol/L in patients of BMI ≥ 28 kg/m^2^.

These findings help resolve conflicting literature on SUA-BMD relationships. While some studies reported protective associations in general populations [32–34], others showed no correlations [35] or negative associations [19] in specific subgroups. In T2D cohorts, similar discrepancies existed with positive correlations in postmenopausal women [36] contrasting with our findings in non-overweight men. The observed heterogeneity likely stemmed from population differences, particularly regarding BMI stratification. Our analysis revealed that the SUA-BMD relationship was BMI-dependent in men with T2D, potentially explaining why previous studies with different population characteristics yielded inconsistent results.

The BMI-specific effects may arise from dual biological roles of uric acid. In BMI < 24 kg/m^2^group, higher SUA (420–479 μmol/L) was associated with greater TLBMD, potentially reflecting uric acid's antioxidant protection against osteoclast activation and ROS-mediated bone loss [37–39]. In individuals of BMI 24–27.999 kg/m^2^, the SUA (360–419 μmol/L) level showed optimal BMD, suggesting a balance between antioxidant benefits and avoidance of hyperuricemia-related risks. The absence of association in obese patients may stem from several factors: chronic inflammation overriding antioxidant benefits [40, 41], altered renal uric acid handling [42], dominance of mechanical loading effects on BMD [43, 44], and the non-linear SUA-BMI relationship altering SUA's biological impact in severe obesity [45]. Furthermore, lean mass as a mediator of BMD through mechanical loading and cytokine production [46] likely interacts differently with SUA across BMI categories.

These results suggested BMI-stratified SUA management may optimize bone health in T2D. For patients of BMI 24–27.999 kg/m^2^, Maintaining SUA at 360–420 μmol/L may benefit lumbar BMD. Current urate-lowering therapy (ULT) targets (typically < 360 μmol/L) might require reconsideration in this group when preserving BMD is prioritized, potentially supporting slightly higher SUA thresholds. In patients of BMI < 24 kg/m^2^, higher SUA (420–479 μmol/L) was associated with better TLBMD, suggesting caution with aggressive ULT that might compromise BMD. For the patients of BMI ≥ 28 kg/m^2^, BMD management should prioritize weight loss and glycemic control over SUA modulation given the minimal association observed. Clinicians should weigh potential skeletal benefits against risks of gout and cardiorenal disease [47], avoiding overtreatment that might negate uric acid's antioxidant role [48].

The result may have significant clinical implications. Firstly, a positive correlation was observed between SUA levels and BMD. However, it was important to note that high SUA levels may not be beneficial, especially in overweight and obese patients with T2D. Secondly, the optimal SUA level for BMD differed in various BMI levels. This may be valuable for guiding clinical interventions focused on lowering SUA levels for T2D patients. Thirdly, it was evident that the baseline SUA levels held predictive value in assessing BMD in patients with T2D.

This study highlighted several important advantages. Firstly, the research subjects were rigorously selected. Secondly, the relationship between SUA and BMD was rigorously tested using three different models, adjusting for a range of potential confounding variables including BMI, LDL, 25(OH)D, H, ALP, age, FINS, FPG, Ca, duration of T2D, TG, Trunk/LimbFr, 2hCpeptide, 2hPG, SCr, AG, FCpeptide, 2hINS, HbA1c, TFr, Hipline, TC, TLM, Trunk/LegsFr, Waistline, TFM, and Wt. Furthermore, the observed correlation between SUA and BMD varied across different BMI, which might help explain the controversies regarding this relationship in previous studies.

We should also consider some limitations. Firstly, a cross-sectional study design tends to only constrain to assessing associations but uncertainty concerning the temporal relationship of exposure-outcome. Secondly, the participants were restricted to men aged ≥ 50 years. Thus, these findings might not be generalized to men aged below 50, or women.

## Conclusion

SUA levels showed BMI-dependent associations with lumbar BMD in T2D, being positively correlated in overweight men with an optimal range of 360–420 μmol/L. Further research is required to clarify a clearer correlation between them.

## Data Availability

The data used to support the findings of this study are available from the corresponding author upon request.
